# Screening of post-mortem tissue donors for *Coxiella burnetii* infection after large outbreaks of Q fever in The Netherlands

**DOI:** 10.1186/1471-2334-14-6

**Published:** 2014-01-06

**Authors:** Marja J van Wijk, D Willemijn Maas, Nicole HM Renders, Mirjam HA Hermans, Hans L Zaaijer, Boris M Hogema

**Affiliations:** 1 BISLIFE Foundation, PO Box 309, 2333BD Leiden, The Netherlands; 2 Regional Laboratory Medical Microbiology and Infection Prevention, Jeroen Bosch Hospital, PO Box 90153, 5200ME ‘s Hertogenbosch, The Netherlands; 3 Department of Blood-borne Infections and Viral Diagnostic Services, Sanquin blood supply foundation, PO Box 9892, 1006AN Amsterdam, The Netherlands; 4 Department of Clinical Virology (CINIMA), Academic Medical Center, PO Box 22660, 1100DD Amsterdam, The Netherlands

**Keywords:** Coxiella burnetii, Q fever, Tissue transplantation, Serological screening, Zoonotic infections, Outbreaks

## Abstract

**Background:**

After the largest outbreaks of Q fever ever recorded in history occurred in the Netherlands, concern arose that Coxiella may be transmitted via donated tissues of latent or chronically infected donors. The Dutch Health Council recently advised to screen tissue donors, donating high risk tissues, for Coxiella infection.

**Methods:**

After validation of an enzyme immunoassay (EIA) test for IgG antibodies against phase 2 of *C. burnetii* for use on post-mortem samples, serum samples of 1033 consecutive Dutch post-mortem tissue donors were tested for IgG antibodies against phase 2 of *C. burnetii*. Confirmation of reactive results was done by immunofluorescence assay (IFA). All available tissues (corneas, heart valves, skin and bone marrow) from donors with IgG reactivity were tested for presence of Coxiella DNA by PCR. Risk factors for IgG reactivity were investigated.

**Results:**

After validation of the tests for use on post-mortem samples, 50/1033 donors (4.8%) screened positive for phase 2 anti-Coxiella IgG by EIA, and 31 were confirmed by IFA (3.0%). One donor showed a serological profile compatible with chronic infection. All tested tissues (25 corneas, 6 heart valves, 4 skin and 3 bone marrow) from donors with IgG reactivity tested negative for the presence of Coxiella DNA. Except for living in a postal code area with a high number of Q fever notifications, no risk factors for IgG reactivity were found.

**Conclusions:**

The strong correlation between notifications and seroprevalence confirms that the used assays are sufficiently specific for use on post-mortem samples, although one has to be aware of differences between batches. Thus, this study provides a validated method for screening tissue donors for infection with *Coxiella burnetii* that can be used in future outbreaks.

## Background

Q fever is a zoonotic disease caused by infection with the bacterium *Coxiella burnetii*. Airborne transmission from infected goats and sheep is the principle mode of transmission to humans. In the Netherlands Q fever outbreaks started in 2007 and increased in the subsequent years. Over 4000 cases and 25 casualties were reported between 2007 and 2010, making it the largest Q fever epidemic ever reported. After various measures were taken, including culling of pregnant goats at infected farms, the number of reported cases declined in 2010 and returned to less than 100 cases per year in 2011.

Most Coxiella infections pass asymptomatic (± 60%), but mild flu-like illness or severe disease with pneumonia or hepatitis occurs. Current data suggest that 1.5 to 2% of infected persons develop chronic Q fever, most often persons with underlying (cardiovascular) disease or immunocompromised individuals [1]. Chronic Q fever often presents as endocarditis, but currently in the Netherlands many cases of vascular infections occur [2]. Estimates of the case fatality rate for chronic Q fever vary from 5 to 50%, depending on clinical manifestations and treatment options [3].

In literature, no *C. burnetii* transmission through tissue transplantation has been described, but single cases of transmission through blood transfusion [4] and through bone marrow transplantation to an immunocompromised recipient [5] were reported as well as transmission through organ transplantation in animals [6]. *C. burnetii* has been shown to persist in various tissues after acute infection, most notably in bone marrow [7,8]. *C. burnetii* will not be detected by microbiological cultures, as employed in tissue banks. A pilot study indicated that serological testing on post-mortem blood is possible, with the test showing sufficient sensitivity and specificity to proceed to a larger scale of testing. *C. burnetii* is not rendered harmless by storage, even at low temperatures, but some processing techniques may have a sterilising effect, e.g. glycerolisation, alcoholisation, peracetic acid treatment, irradiation or decellularisation. Different tissues may pose different risks, with heart valves and bone (in which Coxiella may grow during chronic infection) probably posing the highest risk [9]. The tissue with the lowest transmission risk through transplantation is probably cornea, which is avascular, and no Q fever symptoms have been described in the anterior eye [9].

With progression of the Dutch outbreak, the risk of transmitting *C. burnetii* through tissue transplantation was minimized by implementing control measures based on a risk assessment [9]. These included exclusion of donors with increased risk of acute or chronic Q fever, based on occupational and geographical risk factors, and on clinical presentation and medical history. Furthermore, the possibility for serological testing was investigated as well as processing strategies that can render the bacteria harmless. Currently, the Dutch Health Council has advised to screen tissue donors (with the exception of cornea donors) for signs of current or past infection with *C. burnetii*, the major concern after the outbreaks being that tissues from donors with chronic Q fever might be infectious [10]. The current study was undertaken prior to this advice with the following aims:

•Validation of a serological test for use with post-mortem samples and estimation of the seroprevalence of *C. burnetii* infection in Dutch tissue donors.

•To determine whether *C. burnetii* DNA can be detected in tissues for transplantation (cornea with scleral rim, skin, heart valves, bone marrow) after *Coxiella* infection.

This study provides information on which donor selection policies for tissue transplantation can be based, with an optimal balance between donor safety and tissue availability, providing a screening system that can be used during outbreaks of Q fever.

## Methods

### Donor serum samples

Serum samples were included from all Dutch post-mortem tissue donors between October 2010 and June 2011, from whom at least one tissue was approved at initial assessment. All serum samples were obtained within 24 hours post-mortem, unless there was haemodilution or insufficient quality, in which cases (pre-transfusion) ante-mortem samples were used for testing. Standard donor selection criteria were applied during the study. Regarding Q fever the following donors were excluded: donors with proven acute Q fever; donors with signs of acute Q fever (such as flu-like symptoms, pneumonia without a clear cause or identified pathogen or hepatitis); and donors with a high risk of acute or chronic Q fever, such as donors with occupational hazard (i.e. farmers and veterinarians).

### Donor tissue samples

Tissue samples from all donors who tested positive for IgG antibodies against phase 2 of C. *burnetii* were collected and stored for detection of Coxiella DNA (provided that permission for transplantation-related research had been given).

### Detection of Coxiella antibodies

Serum samples from the post-mortem tissue donors were tested for IgG antibodies against phase 2 of *C. burnetii* using the CE-marked Serion enzyme immunoassay (EIA) test (Serion, Clindia Benelux, Leusden, the Netherlands). The cut-off values for EIA (borderline) positivity were determined according to the manufacturer’s instructions. Borderline reactive samples were considered positive. Confirmation of positive samples was performed using an immunofluorescence assay (IFA) for IgG antibodies against phase 1 and 2 of C. *burnetii* (Focus Diagnostics, Cypress, CA) following instructions of the manufacturer, using a cutoff dilution of 1:32. An IgG phase 1 antibody titer ≥ 1/1024 was considered suspect for chronic Q fever [11-13]. Both serologic tests have, to our knowledge, never been used for post-mortem blood samples. Cadaveric specimens are of lower quality than regular blood samples, showing more false-positive and false-negative reactions. Validation of the EIA and the IFA was therefore performed prior to the start of the study.

The Serion anti-Coxiella phase 2 IgG EIA was validated by testing 45 randomly selected donated post-mortem samples. One of the 45 samples tested positive; this result was confirmed by IFA. The average EIA signal (measured as the optical density signal to cutoff ratio) was not different between negative post-mortem samples and negative samples from 92 healthy blood donors from the Northwestern part of the country (OD/CO 0.107 ± 0.112 versus 0.104 ± 0.008, *p* = 0.87). The clinical specificity in post-mortem samples could not be determined, since no samples from post-mortem tissue donors historically proven to be reactive were available.

Effects of the cadaveric nature of the samples on the sensitivity were assessed by spiking samples. A panel of 20 samples from post-mortem tissue donors, showing various degrees of haemolysis, was spiked with 1/8 volume of serum from a healthy blood donor who tested positive for both anti-Coxiella phase 1 and 2 IgG [14]. The average increase in OD caused by the spiking was slightly higher for post-mortem samples than for sera from 18 healthy blood donors (ΔOD = 0.556 ± 0.075 versus 0.486 ± 0.080, respectively; p = 0.009). The signal increase was not significantly different in hemolytic samples compared to normal post-mortem samples, suggesting the EIA test is sufficiently robust for measuring post-mortem samples of relatively low quality.

A small-scale validation of the IFA for measuring post-mortem serum samples was performed by measuring 27 random post-mortem samples. All samples tested negative and no high background fluorescence was observed. Further validation was done by spiking samples suspected to be false-positive in the EIA (see results section). 18 samples with a varying degree of EIA reactivity that were not confirmed by IFA were spiked with 1/8 volume of serum from a healthy blood donor positive for phase 1 and 2 IgG. The fluorescence intensity after spiking did not significantly differ between EIA-negative samples, borderline-positive samples and IEA-positive samples, suggesting that the negative IFA results (of the unspiked samples) were not caused by signal inhibition.

### Detection of Coxiella DNA

Donated tissues from donors positive for *C. burnetii* antibodies were tested for the presence of *C. burnetii* DNA by PCR. Corneas were frozen at −20°C until analysis as soon as serology results were known. For cornea donors, a wedge of cornea, including scleral rim, was used for DNA extraction and PCR testing. For skin donors, skin samples, frozen in an 85% glycerol buffer, and skin storage solution were tested. For heart valve donors, aortic and pulmonary valves, aortic and pulmonary artery wall and myocardium were tested. If available, samples were used from cryopreserved grafts, stored for transplantation. If no grafts for transplantation were available, sampling was done from formaldehyde-fixed remnants of the heart that are routinely stored for histological examination. These samples were embedded in paraffin and cut into ribbons before DNA extraction. For musculoskeletal tissue donors, bone marrow samples were taken at retrieval and stored at −20°C for PCR testing.

To efficiently extract DNA from tissues, proteinase K digestion was performed prior to DNA extraction. One fourth of the cornea, ±50-100 mm3 of skin tissue, heart valve tissue or bone marrow was digested with 25 μL of proteinase K (20 mg/ml; Roche Diagnostics GmbH, Mannheim, Germany) and 225 μl digestion solution (0,5% SDS, 21 mM Tris–HCl). Paraffin-embedded tissues were cut into sections. Approximately 1–1.5 cm^2^ of sectioned tissue was put in 250 μL proteinase K digestion solution. Digestions were performed in a thermoshaker at 55°C overnight at 1400 rpm. The subsequent day DNA was extracted using a NucliSens EasyMAG extraction system (bioMérieux, Boxtel, Netherlands). Samples were processed according to manufacturer’s instructions and eluted in 60 μL elution buffer. Ten μL of DNA isolate was added to the PCR, which was performed as previously described [15]. An internal control was added to each sample before EasyMAG isolation and a real-time PCR to detect the internal control target was run parallel to the *C. burnetii* PCR to monitor DNA extraction and PCR inhibition [15]. For cornea samples and paraffin-embedded samples an additional PCR to detect human albumin DNA was performed to ensure sufficient input material in each PCR. Albumin PCR cycle threshold values for cornea PCR’s varied from 19 to 20.3 and for paraffin-embedded tissues from 26.4 to 28.9.

### Data collection

Donor characteristics, such as age, gender, cause of death, clinical characteristics, and place of residence were recorded. Three criteria for increased geographical risk of Q fever were applied. First, living in a four-digit postal code area where at least one Q fever patient was reported in the preceding 3 months. Second, living within a five-kilometre radius of an infected farm, where *C. burnetii* was detected in the bulk tank milk. Third, living in a three-digit postal code area in which the Q fever incidence was higher than 20/100.000 inhabitants in any of the years 2007 to 2010. Approximately 15% of the Dutch population lives in this area, where 86.6% of the Q fever cases were reported. The data on Q fever incidence were obtained from the Dutch National Institute for Public Health and the Environment. The data on bulk tank milk positive farms were obtained from the Dutch Food and Consumer Product Safety Authority. The five-kilometer radius from infected farms to the residence of each donor was determined by measuring the distance between both postal codes.

For all donors who tested positive for *C. burnetii* antibodies, additional clinical, occupational, geographical and -if available- histological data were gathered, to determine the likelihood of previous infection with *C. burnetii,* and to establish the presence of signs of chronic Q fever. Information was gathered by reviewing charts, interviewing the general practitioner or next of kin and by reviewing autopsy results and results of histological examination of remnant hearts after heart valve donation, if performed. Factors that were considered risk factors for chronic Q fever are heart valve disease, heart valve or vascular prosthetics, aortic aneurysms and being immunocompromised [16].

### Ethics

Consent for donation, including testing for transmittable infectious diseases, was obtained prior to donation from either the donor, as registered in the Dutch donor registry, or from the legal next of kin. Because of the Q fever outbreak in The Netherlands the Dutch Health Advisory board deemed additional testing for *C. burnetii* necessary. Since consent for testing for transmittable infectious diseases was obtained and all data were analysed anonymized no specific approval by an Ethical Committee was needed for this study according to the Code of Conduct for Health Research, as implemented by the Dutch Federation of Biomedical Scientific Societies, which is followed by the involved organisations.

### Data analysis

All data were entered in a database and analysed with the statistical software program SPSS 15.0 for Windows (SPSS Inc., Chicago, IL). For statistical analysis of differences between groups, ANOVA or Chi-squared tests were used, when appropriate. P-values ≤0.05 were considered statistically significant.

## Results

### Donor characteristics

Between October 2010 and June 2011, 1033 consecutive donors donated at least one approved tissue, and were tested for IgG antibodies against phase 2 of *C. burnetii.* The mean age of the included donors was 65.6 ± 12.3 years (range 0–85) and 664 (64.3%) of them were male. 950 Donors donated corneas (92.0%), 187 skin (18.1%), 139 heart valves (13.5%), 13 thoracic aortas (1.3%), and 86 musculoskeletal tissues (8.3%).

Thirty-three donors (3.2%) lived in a postal code area, where in the previous three months a Q fever patient had been reported. Eighty donors (7.7%) lived within a five-kilometre radius of a previously contaminated farm, and 147 (14.3%) donors lived in the high incidence Q fever area.

### Serological testing

**Figure 1:**
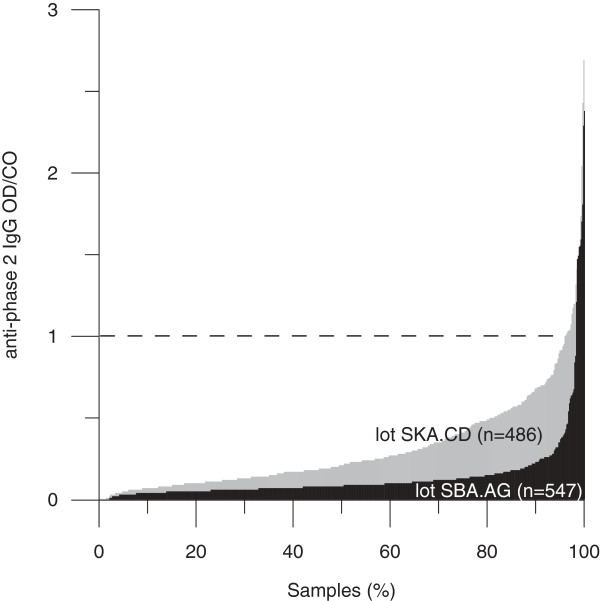
**Distribution of the anti-Coxiella phase 2 IgG signals observed with the two EIA kit lots used in this study.** OD/CO = optical density divided by the cutoff. Samples with OD/CO ≥ 1 are considered positive. The borderline cutoff is lot-dependent and was 0.75 for lot SKA.CD and 0.72 for lot SBA.AG.

Fifty of the 1033 donors (4.8%) tested positive for IgG antibodies against phase 2 of *C. burnetii* by EIA. In 31 donors (3.0%) the reactivity was confirmed by IFA. Twenty-four samples were reactive for both phase 1 and 2, while seven samples were only reactive for phase 2 IgG. One sample showed very high background fluorescence with a few discernible bacteria and was scored positive. One donor (0.1%) had IgG phase 1 and 2 titers of both 1:4096 in the IFA, indicative for chronic Q fever [16]. There was a remarkable difference between the two kit lots used for this study. 12/547 (2.2%) of the donors tested (borderline) positive with the first kit lot (SBA.AG), while 38/486 (7.8%) tested positive with the second lot (SKA.CD, p < 0.001). All donors for whom IgG reactivity was not confirmed by IFA had been tested with the second lot. Usually IFA is more sensitive [17]. Thus, it is seems highly likely that the EIA results were false-positive. Spiking experiments confirmed this (see Methods section). Comparing signals from all measured samples showed elevated background signals for lot SKA.CD and increased numbers of borderline and positive samples using this lot (Figure 1).

These data suggest a problem with cross-reactivity with unknown antigens leading to false-positive reactivity. Indeed, re-testing the 19 discordant samples with a new lot that became available after the study showed ten non-reactive, eight borderline and one positive sample in this lot. Based on these results, we consider only donors who tested positive in both EIA and IFA *C. burnetii* seropositive.

### PCR testing

For 29 of the 31 donors who tested positive in the EIA and IFA one or more tissues were available for PCR testing. For 2 donors the tissues were discarded after initial assessment at the bank for quality reasons. The number of available tissues for PCR testing and the results of PCR testing are presented for each type of tissue in Table 1. No Coxiella DNA was detected in any of the samples.

**Table 1:** **Results of PCR testing for the presence of *C. burnetii *** **DNA in donated tissues**

	**Cornea**	**Skin**	**Heart valves**	**Musculoskeletal**
**EIA and IFA positive N = 31**				
Donated tissues	28	6	5	4
Available for PCR testing	25	6	4	3
PCR negative	24 +1^*a*^	6	4^*b*^	3

Results of PCR testing for the presence of *C. burnetii* DNA in donated tissues in donors who tested anti-Coxiella positive in EIA and IFA.

^*a*^*+1 indicates that one test gave an invalid result due to inhibition of the PCR.*

^*b*^*From one patient no aortic valve and aortic wall tissue was available for PCR testing.*

### Characteristics of donors who tested positive for C. burnetii phase 2 IgG

The mean age in IFA-confirmed seropositive donors was 67.0 ± 9.9 years (range 46–83 years). In the age group from 40–59 years 2.4% of the donors was seropositive, in the group from 60–79 years 3.2% and in group from 80–85 years 3.9%. 22/31 seropositive donors were male (71.0%). The donated tissues are shown in Table 1. Five donors had risk factors for chronic Q fever, namely vascular prosthesis (N = 1), heart valve disease (N = 1), artificial heart valve (N = 1), aortic dissection/aneurysm (N = 2). None of these donors with risk factors for chronic Q fever had a serological profile indicating chronic disease, and in two donors autopsy was performed, which showed no abnormalities. In four seropositive donors the remnant hearts after heart valve donation were examined, which resulted in identification of one donor with a focal pericarditis. However, PCR testing of samples of the heart of this donor did not reveal any *C. burnetii* DNA. The donor who had a serological profile suspect for chronic Q fever was a male donor, 68 years old, who died of an acute cardiac arrest due to a myocardial infarction. The donor did not live in a high risk area for Q fever and had no work related risk factors for Q fever, signs of past infection or risk factors for chronic infection. No autopsy was performed. He donated corneas and skin, which tested negative for *C. burnetii* DNA. No seropositive patients lived in an area where in the last 13 weeks an acute Q fever patient had been reported, but 6 seropositive donors lived within a five-kilometer radius of an infected farm. Eleven donors (35.5%) lived in a high incidence Q fever area.

There was no significant difference in age between groups of seropositive or seronegative donors. There was no significant correlation between gender and seropositivity; the percentage of males was 71.0% in seropositive versus 64.1% in seronegative donors, respectively. There was no increased level of seropositivity in donors living in areas where in the previous 13 weeks acute Q fever patients had been reported or in donors living within a five-kilometer radius from an infected farm. In contrast, the percentage seropositivity was higher in donors living in high incidence Q fever areas as compared to donors living elsewhere (7.5% vs. 2.3%, P = 0.002).

## Discussion

A few years after the large Q fever outbreaks in the Netherlands, concern about potential transmission through tissue transplantation is more focused on donors with silently incubating chronic Q fever than on acute infections. Risk assessments are hampered by limited knowledge about the magnitude of the outbreaks. Although the outbreaks have been studied extensively, most studies were limited to the outbreak areas. To our knowledge, no nation-wide data are available on the prevalence of antibodies against Coxiella after the outbreaks, and our study provides the first estimate of the general seroprevalence of 3.0% (31/1033 donors). It should be added that the cohort may not be representative for the general population. The seroprevalence of 3.0% is twice as high as the prevalence prior to the series of outbreaks which was reported to be 1.5% in the general population, using the same EIA assay [18] and would even be higher if only EIA reactivity was considered (50/1033, 4.8%).

The seroprevalence was 7.5% (11/147 donors) in the area with a high number of Q fever notifications (>20/100.000 inhabitants during the outbreaks). The prevalence in this high-risk area is lower than the previously reported 12% in healthy blood donors in the outbreak area [14] and the 10.7% reported by a hospital in the center of the outbreak [19]. In both cases the most likely explanation for the lower prevalence found among tissue donors is the definition of a larger high-risk area.

In the age group of most donors (40 and older) in samples from 2006–2007 the prevalence was 2.1%. For this age group, the difference with the seroprevalence determined in this study is not significant. Prior to the outbreaks, the anti-Coxiella seroprevalence in the Netherlands was shown to increase with age [18]. A likely explanation why age was not identified as a risk factor for tissue donors is that few post-mortem tissue donors are younger than 40, and during the outbreaks it was unlikely that age affected the exposure risk since Q fever is an airborne infection.

There was an unexpected difference in specificity between the two EIA kit lots used in this study. It is not known whether the high number of false-positive reactions observed with one lot was partly due to the cadaveric nature of the specimens. The intended use of the EIA is the detection of recent or chronic Coxiella infections and not the screening of donors, in whom the low incidence intrinsically leads to a lower positive predictive value. Tests used for screening of donors require a more stringent batch release than regular in-vitro diagnostics tests, but no Coxiella test is on the market for screening purposes.

After acute Coxiella infection, DNA and antigen can be detected for a long time in several tissues, in particular in bone marrow where even in the absence of serological evidence for chronic infection, DNA can be detected up to decades after infection [7,8]. Animal experiments showed that the DNA-positive bone marrow is not infectious in susceptible mice [8]. In contrast, bone marrow from a patient with Q fever endocarditis was infectious in guinea pigs [20]. We detected no Coxiella DNA in any of the tissues available for PCR testing. The 24 PCR-negative corneas confirmed expectations from a risk assessment that no bacteria are present in corneas after infection with *C. burnetii*. For skin, heart valves and bone marrow the numbers of tested donors were quite small and no final conclusions can be drawn. The current study contained only one donor (0.1%) with a serological profile indicating chronic Q fever. The cornea and skin samples of this donor did not test positive for Coxiella DNA. It was not possible to confirm the diagnosis any further since the donor was deceased.

Our data show that the combination of the Serion EIA and confirmation with IFA is an effective algorithm for screening post-mortem samples from tissue donors. The IFA test is considered the ‘golden standard’ for Q fever diagnostics, and is more sensitive than the EIA [17]. The EIA was chosen for the screening because it is less labour-intensive and more convenient for screening larger numbers of samples. It is unlikely that the lower sensitivity of the EIA (as compared to the IFA) increases the risk of transplanting infected tissues, since all patients suffering from chronic Coxiella infection show high IgG titers, and the performance of the test during or shortly after acute infection is excellent. The major disadvantage of using a less sensitive screening test is that the prevalence of Q fever may be underestimated due to waning of the IgG signal [18]. This underestimation will increase over time, assuming no new outbreaks occur. With the current study, that was undertaken at the end of/right after the outbreak period, this underestimation is expected to be limited. An obvious advantage of a less sensitive screening test is that fewer tissues will be rejected based on screening results. Since Coxiella transmission is not expected years after acute infection, using the EIA as a screening method may provide the optimal balance between safety and availability of donated tissues.

After the Q fever outbreaks in The Netherlands the Health Council of the Netherlands advised to test tissue donors donating tissues with a higher risk of transmission for contamination with *C. burnetii*[10]. Because of the geographic spread with cases in almost the whole country in the course of the outbreak, the screening was performed nationwide. The necessity of a nationwide screening was confirmed by the results of this current study in which more than half of the seropositive donors (17 of 31) lived outside the risk areas for Q fever. No guideline was given by the Health Council as to when testing of donors can be stopped. The interval between initial infection and when chronic Q fever becomes manifest is reported to be years [13]. Since the main concern is transmission through tissues from donors with chronic Q fever, it may be reasonable to stop testing when the number of new chronic Q fever patients in The Netherlands drops back to pre-outbreak levels.

## Conclusions

This study provides a validated and effective test algorithm that can be used for the screening of post-mortem samples of tissue donors for antibodies against Coxiella burnetii. Furthermore, this study provides a first estimate of 3.0% of the seroprevalence of antibodies against Coxiella in the Dutch deceased tissue donor population after the recent outbreaks of Q fever in The Netherlands. The fact that all corneas from donors with IgG reactivity tested negative for the presence of Coxiella DNA confirmed expectations from a risk assessment that no bacteria are present in corneas after infection with *C. burnetii*. The data provided by this study can be used to optimize the balance between safety and availability of donated tissues.

## Abbreviations

EIA: Enzyme immunoassay; IFA: Immunofluorescence assay; IgG: Immunoglobulin G; DNA: Desoxyribonucleic acid; PCR: Polymerase chain reaction; C. burnetii: Coxiella burnetii; OD: Optical density; CO: Cutoff; ΔOD: Change in optical density; SDS: Sodium dodecyl sulphate; Tris–HCl: Trisaminomethane hydrochloride.

## Competing interests

There are no financial or non-financial competing interests related to this manuscript to declare.

## Authors’ contributions

MJVW, DWM, NHMR, MHAH, HLZ and BMH were involved in the design of the study. NHMR and MHAH were involved in the molecular analyses. HLZ and BMH were involved in the development of serological testing and BMH was in charge of the serological testing. MJVW, DWM, BMH collected the data, analysed them, interpreted the results and drafted the manuscript. All authors reviewed and revised the manuscript and approved of the final version.

## Pre-publication history

The pre-publication history for this paper can be accessed here:

http://www.biomedcentral.com/1471-2334/14/6/prepub
